# Supplementary material for: Are carbon emissions trading and green financial instruments synergistic? -Comprehensive quantitative research based on content analysis
Source: PLoS One. 2024 Mar 7;19(3):e0298601. doi: 10.1371/journal.pone.0298601 (PMC10919601; doi:10.1371/journal.pone.0298601)
Supplement: S1 Data — (ZIP) [file pone.0298601.s001.zip › Data/地级市Z_F工作报告.zip!/┴╔─■/║∙┬1⁄2╡║╩╨/└┤╫╘╣1⁄2╓┌║┼í╛┬φ┐╦╩2╛▌═°í┐_2017.docx]

各位代表：

　　现在，我代表市人民政府向大会报告工作，请予审议，并请市政协委员和列席会议的同志提出意见。

　　一、2016年工作回顾

　　2016年，是“十三五”开局之年。面对经济下行压力，在市委坚强领导下，在市人大、市政协监督支持下，全市上下团结拼搏，真抓实干，保持了经济社会平稳健康发展。

　　预计2016年，地区生产总值721亿元，增长0.3%;规模以上工业增加值120亿元，下降9%;固定资产投资176亿元，下降7.4%;社会消费品零售总额469亿元，增长8%;一般公共预算收入60.7亿元，增长6.3%;城镇居民人均可支配收入26378元，增长6.5%;农村居民人均可支配收入10949元，增长7%。地区生产总值、固定资产投资、一般公共预算收入增速始终保持全省前列。

(一)重点项目顺利推进

　　深入开展“项目年”活动，实施亿元以上重点项目175个，当年完成投资120亿元。忠旺铝业等106个续建项目有序推进，高桥风电等69个项目开工建设。绥中综合物流园等40个项目建成投产。

(二)农业结构不断优化

　　农业生产喜获丰收，粮食、蔬菜、水果、肉蛋奶产量分别实现100万吨、293万吨、122万吨、43万吨。调整种植业结构，新增粮油和经济作物20万亩。打造果业品牌，更新改造果园7万亩。发展设施农业，新增设施农业3000亩，建成农业精品示范园117个。推进现代渔业建设，水产品产量50万吨，渔业经济总产值59亿元。深化农村改革，完成土地确权268万亩，土地流转面积增长45%。

(三)工业质量逐步改善

　　支持企业增产扩能，实施千万元以上工业项目62个，总投资670亿元。加大企业技改力度，实施技改项目21个，总投资34亿元。落实《中国制造2025葫芦岛行动计划》，实施“制造强市”支撑项目10个，总投资174亿元。鼓励中小企业自主创新，培育科技型企业51家、省级技术中心19个。

(四)第三产业快速发展

　　商贸流通体系建设进一步加快，华联商厦等大型商业设施落户我市，兴隆大家庭等重点项目开工建设。电子商务发展迅速，交易额实现80亿元，增长33%，我市成为全省首家“农村淘宝”上线城市。会展经济效益初显，交易额12亿元，增长8%。金融业不断发展，新增金融机构3家，新增社会融资130亿元，增长8.3%。

　　旅游产业提质升级。启动实施“六个一”工程，全市新增景点14处、温泉度假区9处、民俗博物馆13处、高标准农家乐13处、特色小镇8个、旅游纪念品49种。加快旅游基础设施建设，新建旅游交通干线151公里。加强旅游市场监管，提高旅游服务质量，旅游总收入167亿元，增长16%。

(五)改革开放全面深化

　　持续推进“放管服”改革，成立市行政审批服务局。调整市级行政职权49项，全面开展“双随机、一公开”监管改革。理顺县区、园区管理体制。完成公务用车制度改革。深化国资国企改革，推进中冶有色股权改造，完成6户央企“三供一业”移交。

　　加强区域合作，与秦皇岛签订区域合作协议，与天津大学签订"1+N"市校合作协议，承接京津冀产业转移项目42个，完成投资32亿元。积极开展招商引资，引进省外内资56亿元，签约项目33个;实际利用外资3300万美元，新批外资项目7个。着力扩大出口，扶持外贸综合服务企业3家，培育重点跨境电商平台2个，建设海外仓43个，外贸出口5亿美元。

(六)社会事业协调发展

　　着力解决民生问题。十大民生工程按年度计划推进，完成老旧小区改造、暖房子工程等民生实事23件。拆除10吨以下燃煤锅炉41台，淘汰黄标车8443辆。完成棚户区改造4264户、农村危房改造1245户、建设保障性住房600户、灾后重建房屋3169户。

　　社保体系日趋完善。全市民生支出149亿元，占一般公共预算支出的80.1%。新增城镇实名制就业1.4万人，城镇登记失业率5%，低于省控指标。城乡低保标准分别提高7%和15%，城乡低保取暖救助实现全覆盖。扎实推进脱贫攻坚，落实产业脱贫项目157个，4.9万贫困人口稳定脱贫，61个省定贫困村脱贫销号。

　　各项事业不断发展。完成64所标准化学校建设任务，兴城通过国家级义务教育均衡发展验收，连山、龙港两所大型公益性幼儿园开工建设。市结核病防治所综合楼交付使用，市妇幼保健中心、食品药品检验中心主体完工，正业健康产业园开工建设。市120急救中心通过国际标准认证。市博物馆正式投入使用。累计培训红十字急救员4万人。承办全国拳击锦标赛等5项国家级赛事。

　　社会保持和谐稳定。持续开展专项整治，安全生产形势总体平稳。加快立体化社会治安防控体系建设，扎实开展平安葫芦岛创建，连续获得“全国社会治安综合治理优秀市”称号。双拥工作取得丰硕成果，荣获全国双拥模范城“五连冠”。推进全国精神文明城市创建工作，城市文明水平不断提升。全力做好信访维稳，一批信访积案得到有效化解。成功抵御历史罕见洪灾，安全转移群众23万人次，得到国务院和省委省政府领导充分肯定。

(七)政府建设得到加强

　　认真贯彻落实党的十八届六中全会精神，深入开展“两学一做”学习教育，全面从严治党，规范党内政治生活，政府系统党建工作不断强化。加强民主法治建设，认真落实市委决策部署，主动接受市人大、市政协监督，人大代表建议、政协提案办复率100%、满意和基本满意率99%以上。开展“双评”活动，政府作风进一步转变。

　　各位代表，过去的一年，面对压力和挑战，全市经济社会事业保持平稳发展，这是全市人民共同奋斗的结果。在此，我代表市政府，向全市各族人民，向人大代表、政协委员，向老领导、老同志，向各民主党派、工商联、无党派人士、人民团体、社会各界，向驻葫部队指战员、武警官兵、中省直单位，表示崇高的敬意和衷心的感谢!

　　回顾过去一年工作，我们也清醒地看到，我市发展仍然面临许多困难和挑战。主要表现在：一是经济总量小，民营经济比重低。二是改革步伐缓慢，体制性弊端没有根本解决。三是经济外向度低，港口拉动力弱。四是综合债务率高，债务负担沉重。五是人才短缺，创新能力不足。六是政府职能转变不到位，营商环境亟待改善。对于这些问题，我们将深入调查研究，制定措施办法，以问题为导向，在今后几年逐一破解。

　　二、2017年工作安排

　　今年政府工作的指导思想是：全面贯彻党的十八大和十八届三中、四中、五中、六中全会精神，深入学习贯彻习近平总书记系列重要讲话精神，坚决维护以习近平同志为核心的党中央权威和集中统一领导，牢固树立政治意识、大局意识、核心意识、看齐意识，认真落实中央经济工作会议、省第十二次党代会、市第五次党代会精神，统筹推进五位一体总体布局，协调推进四个全面战略布局，持之以恒落实五大理念和四个着力，坚持稳中求进工作总基调，以深化供给侧结构性改革为主线，以提高发展质量和效益为中心，以振兴实体经济为重点，深入实施五大战略，加快推进七个突破，努力建设生态宜居美丽富庶葫芦岛，以优异成绩迎接党的十九大胜利召开。

　　今年政府工作的奋斗目标是，主要经济指标增速超过全省平均水平，为经济总量位次前移打好基础。地区生产总值增长7%;规模以上工业增加值增长7.5%;固定资产投资增长13%;一般公共预算收入增长5.5%;社会消费品零售总额增长9.5%;城镇居民人均可支配收入增长6.5%;农村居民人均可支配收入增长7.5%。引进内资、利用外资、外贸进出口增幅高于全省平均水平。

　　为实现上述目标，我们将全力做好十个方面工作。

(一)夯实项目基础，拉动有效投资

　　把项目作为经济发展的生命线，持续开展“项目年”活动。实施亿元以上重点项目200个，总投资2100亿元。其中，能源项目19个、工业项目30个、农产品深加工项目7个、服务业项目12个、旅游项目13个、基础设施项目29个、社会事业和房地产等项目90个。落实国家振兴东北政策，向上争取重点项目46个。其中，中冶有色技术改造等传统产业升级项目8个;南票光伏发电等资源枯竭地区转型项目10个;泳装电子商务平台等培育新动能项目15个;中港集团燃气发电等补短板项目13个。结合发展实际，谋划包装重点项目50个。其中，农村电网改造等中央预算内投资项目15个;北京航空航天大学分校等京津冀产业转移项目20个;东戴河全域旅游等产业支撑项目15个。完善领导包扶、协调调度、拉练检查、督查考核机制，强化项目主体责任，加强项目前期保障，优化投资环境，确保项目顺利实施，筑牢发展基础，释放发展动能。

(二)做大做强产业，扩大经济总量

　　深入推进农业供给侧结构性改革。调整粮食与经济作物比例，调减玉米播种面积，发展优质粮油、经济作物30万亩。大力发展设施农业，新增设施农业2万亩。提高畜牧养殖和渔业发展水平，推进畜禽规模化健康养殖，集约化经营，肉蛋奶总产量增长7%以上，建设海洋牧场12万亩。打造品牌果业，新栽果树6万亩，改造果园6万亩。壮大农产品深加工产业，建设兴城农产品加工集聚区，培育省级龙头企业4个、市级龙头企业6个。培育农业新型经营主体，成立市级示范专业合作社30个、市级示范家庭农场30个。完善农产品流通体系，建设农产品批发市场3个，打造京津冀农产品供应基地。

　　做大工业经济总量。坚持稳增长与调结构并重，做强存量，做大增量，增加有效供给，提升发展质量和效益。实施“工业强基工程”，以锦西石化汽柴油改造升级、宏跃北方铜业等项目为拉动，提升先进装备制造业比重，延长石油化工产业链条，推进有色金属精深加工，做大做强支柱产业。实施“产业升级工程”，加快渤船重工核动力平台等10个“制造强市”支撑项目建设，推进首钢东华等20个技改项目，培育壮大一批高端装备制造企业。实施“高新引领工程”，发挥科技企业孵化器示范作用，加快高新区“云启众创”、东戴河“知谷汇”、辽宁财贸学院“双创”基地等创新创业载体建设，大力扶持科技型、成长型中小微企业发展，力争年内入驻项目超过100个。实施“创新增效工程”，完善打渔山泵业等2个公共技术平台，构建有机化工新材料等3个产业技术创新联盟，落实与天津大学、大连理工大学、中科院沈阳分院81个科研成果转化项目。实施“品牌提升工程”，开展质量示范对标活动，打响海工装备、石化成套设备、泵业基地三大品牌，抓质量促管理，创品牌增效益。实施“腾巢换凤工程”，加快招商引资和嫁接改造，盘活现有闲置厂房，集中发展特色加工制造业，使工业经济成为振兴发展强大支撑。

　　推进服务业提质升级。改造提升生活性服务业，支持商贸企业创新营销模式，实现转型发展;积极培育便民新业态，鼓励建设社区便民连锁店，做优传统服务业，提高生活服务品质。大力发展生产性服务业，支持企业与科研机构合作，培育产品研发和技术服务平台;搭建投融资服务平台，建立现货商品交易平台;启动企业上市培育计划，加强政银企合作，合理引导资本投向，有效防范金融风险。做大做强电子商务产业，构建全产业链电子商务体系，交易额突破110亿元，增长35%。积极发展会展经济，交易额突破13亿元，增长9%。加强政策引导，因城因地施策，化解商品房库存，促进房地产市场平稳健康发展。完善现代物流产业，加快建设玉皇商城物流综合体等重点项目，加强供给保障，持续繁荣市场，打造辽西商贸物流枢纽。

　　加快发展旅游产业。继续实施“六个一”工程，重点建设兴城温泉水上乐园等14个旅游项目。完善旅游基础设施，建设旅游交通干线71公里，开通滨海旅游观光巴士，成立旅游公共服务中心。推进景区提质升级，争取银泰水星乐园晋升4A级景区、九门口长城晋升5A级景区，创建兴城、绥中国家级全域旅游示范县。强化区域合作，开拓京津冀、内蒙古和国外市场，健全完善旅游市场网络。举办泳装文化博览会、菩提文化博览会、紫砂文化周等大型节会。加强旅游市场整治，创新管理体制，改善旅游环境，提升旅游发展软实力，打造滨海旅游名城。

　　(三)注重协调发展，推进同城同质

　　优化发展空间，提升城市品质，夯实城市承载。改造老城，提升新城，连接兴城;规划同步、交通同城、设施共建、服务共享，实现三城同盛，打造城市魅力。

　　建成外环路、海航路，规划建设海翔路高架桥，解决新老城区交通瓶颈问题。开工建设高铁站至中央商务区快速干道，打通龙前街，改造海辰路立交桥，完善城市交通路网。改造连山、龙港棚户区，改造老城区管网8万米、老旧小区7个，完成暖房子工程100万平方米;建设老区滨河公园，改造体育休闲广场，逐步改变老城区面貌。完善中央商务区服务功能，建成CBD商业综合体，开工建设城市综合规划展览馆、月亮河海滨栈道，规划海洋游乐馆，建成高铁兴城西客站，建设首山湖温泉度假区，提升城市美化亮化水平，打造亮丽城市名片。

　　提高城市承载力，实施龙湾海滨雨污分流、五里河生态修复工程，推进海绵城市建设。完成青山水库环保工程，市净水厂、杨家杖子净水厂、市水质检测中心投入使用，确保市民喝上放心水。加强生态环境保护，提标改造兴城、绥中、建昌、南票污水处理厂，建设垃圾焚烧发电厂;淘汰20吨以下燃煤锅炉，继续淘汰黄标车，城市道路机扫率达到85%。理顺体制，完善机制，依法精细管理，全面提升城市文明程度。

　　统筹城乡发展，加快新型城镇化建设。维修改造干线公路94公里，新改建农村公路450公里，完善农村基础设施。突出典型示范，发展一批人口集聚、产业集中、设施齐全、功能完善的中心城镇。建设宜居乡村，发展一批产业鲜明、文化独特、风格各异、环境优美的特色小镇。

(四)全面深化改革，释放发展活力

　　深化供给侧结构性改革，制定分类实施方案，完成“三去一降一补”任务。深入推进“放管服”改革，成立市营商环境监督管理局。重新审定政府部门“三定”方案，进一步推进“双随机、一公开”监管改革，在东戴河新区先行试点企业项目承诺制。深入推进国资国企改革，完成央企“三供一业”移交任务，推进厂办大集体改革。加快推进事业单位分类改革，发展壮大企业集团，化解政府性债务，确保全市债务风险安全可控。加快推进财税改革，全面推进预算公开。全面启动公立医院综合改革，满足群众基本医疗服务需求。推进文化旅游体制改革，繁荣文化旅游事业。推进土地“三权分置”改革，扩大农村土地流转规模。深化供销合作社综合改革，提升服务“三农”质量和水平。

(五)加快扩大开放，增强发展动力

　　牢固树立开放意识，以扩大开放为引领，以招商引资为动力，促进区域经济加快发展。

　　狠抓招商引资。实施园区招商，充分利用园区产业基础、生产要素、公共服务等资源优势，发挥招商引资主力军作用。高新区重点围绕先进制造、精细化工、高新技术、新材料产业招商，引进项目8个，引进资金5亿元;北港工业区重点围绕装备制造、临港物流、现代服务业招商，引进项目10个，引进资金20亿元。实施产业链招商，落实八大门类产业发展政策，依托锦西石化、忠旺铝业等优势企业，引进炼化副产品、精细化工、有色金属精深加工等项目20个，引进资金12亿元。对接京津冀招商，引进中非海上浮式发电平台等项目30个，引进资金100亿元。对接央企招商，引进首农集团农产品加工等项目10个。面向日韩、港台等重点国家和地区招商，引进正大集团食品加工等项目14个，新批外资企业12家，实际利用外资3.5亿美元，实现招商引资新突破。

　　着力扩大开放。融入“一带一路”，加快葫芦岛港建设，搭建开放平台，新建绥中港区2个10万吨级码头，启动葫芦岛港疏港铁路工程，加快绥中港区、柳条沟港区综合物流园建设;完善葫芦岛港规划，整合港口资源，推进与营口港务集团战略合作，实现口岸外贸运输正常化。建设海关特殊监管区，吸引域外投资，推进加工贸易企业集群发展;搭建综合跨境电子商务平台，加速建设外贸海外仓，扩大进出口贸易;引进培育外贸综合服务企业，提高贸易便利化水平;巩固船舶、成品油等传统优势，扩大农产品、工业消费品出口，实现对外开放新突破。

　　(六)突出区域特色，做强县区经济

　　以园区为载体，扶持实体经济，培育主导产业，壮大县区经济，筑牢县区发展基础。兴城以滨海经济区为依托，培育高新技术产业，大力发展食品加工、旅游和新兴服务业，巩固泳装产业集群，建设高新技术产业基地、世界泳装生产基地和康养胜地。绥中以港口建设为牵动，大力发展临港产业、综合物流业，培育东戴河高新技术、文化旅游产业集群，努力成为蒙、俄出海大通道。建昌依托生态资源优势，发展矿产品精深加工，做优农产品加工业，做强乡村旅游产业，打造绿色有机农副产品生产基地。连山推进老城区改造，提档升级传统服务业，加快发展打渔山园区装备制造、节能环保、新型建材产业，打造东城商业金廊。龙港以中央商务区为重点，以重大商贸项目为依托，建设现代服务业发展示范区。南票和杨家杖子以产业转型为引领，实施独立工矿区改造，大力发展新型建材、紫砂、膨润土精深加工产业，尽快实现转型发展。

　　制定县区经济发展三年滚动计划，明确发展方向，确定发展目标，创新发展模式，因地制宜，错位发展。加强财税政策支持，带动社会资本投向县区经济。加大科研投入，完善人才引进、培养机制，激发县区创新活力。建立考核体系，健全管理制度，调动社会力量，形成发展合力，加速提升县区经济整体实力。

　　(七)优化营商环境，壮大民营经济

　　把民营经济摆在更加突出位置，强化企业主体地位，制定政策措施，集聚要素资源，加快企业集群化发展，着力补齐民营经济短板。

　　推动大众创业、万众创新，建设中小微企业创业基地，大力发展科技企业孵化器、创业园、众创空间等创业载体，积极培育民营经济群体。实施“个转企”“小升规”“大做强”，个体工商户转企650户以上，新增规上工业企业15家、限额以上服务业企业100家，主营业务收入亿元以上工业企业70家，推进民营企业提质升级。围绕主导产业，延伸产业链条，做好配套服务，发展一批上下游民营企业;发展新兴产业，加强校企、院企合作，加快科技成果转化，培育一批科技型民营企业;推进PPP模式，吸引社会资本投入，引进一批域外民营企业;深化国资国企改革，支持民营企业参与并购重组，壮大一批混合所有制企业。完善民营企业服务平台，健全政策体系，强化服务指导，创新融资担保机制，充分发挥工商联、商会协会作用，着力破解民营企业发展难题，做大民营经济总量。

　　(八)保障改善民生，实现共享发展

　　完善社会保障体系。有效防范养老金支付风险，确保养老金及时足额发放。加强就业服务，强化创业引领，新增城镇实名制就业1.2万人，城镇登记失业率控制在5%以内，零就业家庭保持动态为零。有效保障困难群众基本生活，城乡居民低保标准、农村特困人员供养标准平均提高6%以上，资助城乡低保对象、特困人员全部参加医保或新农合。建设县(市)区综合性社会福利中心，完成农村敬老院改造工程。全面开展因病支出型贫困家庭救助，建成市残疾人康复中心。

　　积极发展社会事业。推进义务教育均衡发展，建设省级标准化学校45所，改造农村薄弱学校80所，绥中、连山、杨家杖子通过国家级义务教育均衡发展验收。高度重视学前教育，两所大型公益性幼儿园建成使用。提高高中教育教学水平，解决优质生源外流问题，改建市二高中。大力发展职业教育，扩大中职学校招生规模。建成市特教学校教学楼，特教学校教师津贴提高30%。加快引进、培养美育教师，推动美育教育快速均衡发展。完善基层卫生服务体系，实现基层卫生计生信息化，提升社区卫生机构服务能力。开工建设市康复医院。市结核病防治所、市妇幼保健院投入使用。强化食品药品安全监管，创建兴城、绥中食品安全放心县。加强社会主义精神文明建设，创建全国文明城市。推进军民深度融合发展，扎实做好新一轮双拥模范城创建工作。加快构建现代公共文化服务体系，满足人民群众基本文化需求，建设画家写生基地、北方影视基地。完善全民健身服务体系，推动全民健身事业健康发展。加强文物保护，维修圣水寺，修缮兴城古城，管护好长城。承办国际台球锦标赛、全国自行车联赛、铁人三项赛等重大赛事，丰富市民文化体育生活，提升城市知名度。

　　确保社会安全稳定。严格落实“三个责任”，持续开展专项整治，深刻吸取南票煤矿暗访存在重大安全隐患教训，坚决遏制重特大安全生产事故发生。加强应急管理，重视防灾减灾，提高处置突发公共事件能力。深入推进信访制度改革，切实解决信访突出问题。重视民族宗教工作，维护民族团结、宗教和谐。扎实推进平安葫芦岛创建，创新社会治安防控体系，加快警务云平台、天眼工程建设，让人民群众更有安全感、幸福感、获得感。

(九)全力脱贫攻坚，补齐小康短板

　　落实扶贫政策，推进精准扶贫、精准脱贫，完成省定60个贫困村脱贫销号、4.7万贫困人口稳定脱贫任务。

　　发展设施蔬菜、优质林果、规模养殖等特色产业，提高贫困地区脱贫能力。加快旅游扶贫，重点建设21个全国旅游扶贫示范村。加强项目扶贫，加快建设建昌光伏发电等重点项目，培育一批电商服务站。推进金融扶贫，设立风险补偿金，破解贫困地区融资、担保难题。创新扶贫机制，探索建立“产加销一条龙”扶贫新模式。突出龙头企业、农业合作社牵动作用，积极培训贫困劳动力和致富带头人，带动贫困人口脱贫。实现农村低保制度、残疾人补贴制度与扶贫开发政策有效衔接，保障贫困人口义务教育、基本医疗和住房需求。发挥众帮优势，加强定点帮扶、结对帮扶、村企共建、军民共建，形成扶贫合力，巩固脱贫成果，确保稳定脱贫。

(十)加强政府建设，提高服务水平

　　坚定正确政治方向。牢固树立政治意识、大局意识、核心意识、看齐意识，服从市委领导，自觉接受人大及其常委会依法监督、政协民主监督，履行政府从严治党主体责任，全面加强政府系统党建工作，转变政府工作作风。

　　建设法治诚信政府。牢固树立法治理念，依法行使权力、履行职责。开展第三方评估，对症下药，用硬措施整治软环境。强化规则意识、契约精神，依法依规兑现政府承诺，以诚信服务取信于民，努力提高政府公信力。

　　建设务实高效政府。坚持从实际出发，出实招、办实事、求实效，一张蓝图绘到底。弘扬雷厉风行、敢于担当、真抓实干的作风，不张扬、不冒进、不说空话，哈下腰来，埋头苦干，一抓到底，抓出成效。强化绩效考核，坚决查处为官不为、懒政怠政行为，切实提高政府行政效能。

　　建设清正廉洁政府。严格执行《关于新形势下党内政治生活的若干准则》和《中国共产党党内监督条例》，全面落实“一岗双责”。严肃惩处腐败现象，坚决整治吃拿卡要等不正之风，树立风清气正的政府形象。

　　各位代表，实现葫芦岛新发展、新跨越、新突破，重任在肩，时不我待。让我们更加紧密地团结在以习近平同志为核心的党中央周围，在市委的坚强领导下，抢抓机遇、开拓进取，加快建设生态宜居美丽富庶葫芦岛，为全面建成小康社会而努力奋斗!
